# Modification of Electrical Transport in a Grating-Electrode GaN/AlGaN Quantum-Well Device by Ti_3_C_2_T_x_ MXene Under 254 nm Illumination

**DOI:** 10.3390/nano16191204

**Published:** 2026-09-23

**Authors:** Boning Wei, Xi Wei, Haibing Qiu, Cheng Lei, Fengchao Li, Ting Liang, Jiangang Yu, Wenxian Yang, Shulong Lu, Shan Jin

**Affiliations:** 1 School of Semiconductor and Physics, North University of China, Taiyuan 030051, China; sz202519023@st.nuc.edu.cn (B.W.); s202519045@st.nuc.edu.cn (X.W.);; 2 State Key Laboratory of Widegap Semiconductor Optoelectronic Materials and Technologies, North University of China, Taiyuan 030051, China; 3 Suzhou Institute of Nano-Tech and Nano-Bionics, Chinese Academy of Sciences, Suzhou 215123, China; wxyang2014@sinano.ac.cn (W.Y.);

**Keywords:** Ti_3_C_2_T_x_ MXene, GaN quantum well, AlGaN, ultraviolet photodetector, grating electrode, static photoresponse

## Abstract

Ultraviolet (UV) photodetectors are essential for flame monitoring, environmental sensing, UV photography, optical communication, and biochemical analysis, and III-nitride quantum-well structures are promising platforms for such devices. Two-dimensional Ti_3_C_2_T_x_ MXene, which combines metallic conductivity with a termination-dependent work function, offers an attractive route to modifying the surface and interface transport of GaN-based photodetectors. The purpose of this work is to determine whether integrating a Ti_3_C_2_T_x_ MXene surface region onto a grating-electrode GaN/AlGaN asymmetric coupled-quantum-well UV photodetector selectively enhances the photoresponse or predominantly modifies the overall device conductance. The device was fabricated and assessed under 254 nm illumination. Optical and scanning electron microscopy verified the patterned device and the continuous grating region, and the fabrication sequence explained the integration procedure. Static current–voltage (I–V) characteristics of the same device were measured both before and after Ti_3_C_2_T_x_ MXene coverage. Nonlinear transport and a discernible light–dark current gap were present in both states. Before MXene coverage, the device produced a dark current of 1.98 μA, a net photocurrent of 1.90 μA, and a light-to-dark current ratio of 1.96 at +3 V; after coverage, the corresponding values were 0.837 mA, 0.835 mA, and 2.00. The increase in the dark current was reproduced on three grating-electrode devices, for which MXene coverage raised the dark current by a factor of between about 4 × 10^2^ and 1.3 × 10^3^ depending on the device and the sweep direction, whereas a control structure without the grating electrode remained nearly three orders of magnitude below the MXene-covered devices. Switching the 254 nm source at a fixed bias of +1 V produced reproducible switching between two current levels with a light-to-dark ratio of 2.10–2.15; the transitions were completed within the 0.1 s sampling interval. Ti_3_C_2_T_x_ MXene coverage raised the total current level in both electrical states while maintaining a detectable relative light–dark contrast from +1 to +5 V, indicating that the dominant measured change was an increase in device conductance rather than a selective enhancement of the relative photoresponse. Within the scope of this transport study centered on static I–V measurements and supplemented by a single-device time-resolved measurement, the results demonstrate static separation between the dark and 254 nm-illuminated states and modification of the overall electrical transport by MXene coverage on the same device; they do not claim an improvement in photodetector figures of merit, and absolute figures of merit are not reported because the optical power incident on the device was not calibrated.

## 1. Introduction

Flame monitoring, environmental sensing, UV photography, optical communication, and biochemical research are all applications for ultraviolet (UV) photodetectors. GaN and AlGaN are direct wide-bandgap semiconductors that have strong breakdown fields, thermal and chemical stability, and UV spectrum coverage [1,2,3]. III-nitride heterostructures are appropriate platforms for UV photodetection because of these characteristics.

Ultraviolet photodetectors based on wide-bandgap semiconductors are limited in practice by the trade-off between dark current and responsivity, which is largely set by the contact configuration and by the surface and interface chemistry of the device. Engineering the metal–semiconductor interface of GaN-based devices with conductive two-dimensional materials is therefore an active strategy to tailor carrier collection, contact barriers, and dark current. Understanding how such surface modifications affect the overall transport level—rather than assuming an automatic enhancement of the photoresponse—is essential for the design of MXene-modified UV detectors.

GaN/AlGaN quantum wells offer nanoscale control over carrier transit and confinement. GaN wells and AlGaN barriers have been used to demonstrate multiple quantum-well UV photodetectors, with the electrical response controlled by the carrier-collection geometry and the epitaxial architecture [4,5]. The active GaN/AlGaN platform in the current device is formed by placing 20 periods of asymmetric coupled quantum wells between strongly doped n^+^-GaN contact layers. The 20-period design provides a substantial interaction volume for incident 254 nm photons while remaining compatible with the top-contact and grating geometry, and the asymmetric coupled-well configuration can introduce internal polarization fields [4,5] that may influence carrier transport; the present static measurements do not, however, isolate this contribution to the photoresponse.

The surface terminations of MXenes, which are conductive two-dimensional transition-metal carbides and nitrides, can affect their interfacial transport and work function [6,7]. Ti_3_C_2_T_x_ is the MXene utilized in this work. For UV-sensitive devices, MXene/GaN contacts and junctions have been studied [8,9,10,11]. The illuminated region, transport distance, and carrier-collection boundary are all simultaneously defined by the patterned electrode, so the grating configuration can be designed such that the exposed area serves simultaneously as the illumination window and as the site of MXene deposition. The viability of combining two-dimensional materials with III-nitride platforms is further demonstrated by related graphene/GaN and PtSe2/GaN devices [12,13,14,15], and the termination-dependent work function of Ti_3_C_2_T_x_ makes the barrier height of such contacts tunable [16].

The electrical role of Ti_3_C_2_T_x_ at a GaN surface is governed not only by its high conductivity but also by its termination-dependent work function and the resulting interfacial band bending. Because practical Ti_3_C_2_T_x_ generally contains mixed O-, OH-, and F-related terminations, its contact with n-GaN may range from a rectifying to a more conductive configuration depending on surface chemistry, processing history, and the native state of the GaN surface [17]. Consequently, MXene integration can modify both the junction barrier and the series or contact resistance, and these effects must be separated from changes in photocarrier generation [8,9,10,11]. The interfacial chemistry of the MXene/GaN junction—governed by the MXene terminations and by GaN surface states—is therefore a key design parameter that determines the effective barrier height and the series resistance of the device [18].

Previous MXene-GaN photodetectors have demonstrated that a van der Waals metal–semiconductor contact can suppress dark current and facilitate photocarrier collection when MXene replaces a conventional metal contact [8]. The present comparison is different: Ti_3_C_2_T_x_ is added to the same pre-existing grating-electrode device, and the measured dark current and photocurrent both increase after coverage. The analysis therefore focuses on configuration-dependent transport rather than assuming that the mechanism reported for a different electrode architecture applies unchanged to the present device.

In this work, a grating-electrode GaN/AlGaN asymmetric coupled-quantum-well device is integrated with a Ti_3_C_2_T_x_ MXene surface region. The study is structured around three experimentally supported questions: how the transport and relative light–dark contrast of the same device change after Ti_3_C_2_T_x_ MXene coverage; whether the composite structure can be fabricated; and whether the dark and 254 nm illuminated states can be distinguished electrically. Instead of equating an increase in absolute current with an independent detector-performance boost, the present work is limited to examining whether the dark and 254 nm-illuminated states of the same device can be distinguished in static I–V measurements and how MXene coverage modifies the overall transport; it is framed as a preliminary single-device static transport study.

## 2. Materials and Methods

### 2.1. Structure Design and Device Fabrication

A GaN-based ultraviolet photodetector with a MXene-modified grating electrode was fabricated. Figure 1 shows the schematic diagram of the device structure and its epitaxial structure. The epitaxial structure was grown by metal–organic chemical vapor deposition (MOCVD) on a GaN/sapphire platform using trimethylgallium, trimethylaluminum, and ammonia as precursors and silane as the n-type dopant source. It consists of a 20-period GaN/Al_0.25_Ga_0.75_N asymmetric coupled-quantum-well active region, an approximately 50 nm n^+^-GaN top contact layer with the same nominal carrier concentration, and a roughly 500 nm n^+^-GaN bottom contact layer with a nominal carrier concentration of 1 × 10^19^ cm^−3^. An active-region thickness of roughly 286 nm corresponds to the nominal period thickness of 14.3 nm. Al_0.25_Ga_0.75_N barriers, intrinsic GaN wells, and a 1.3 nm highly doped GaN layer are present in every period. Figure 1b summarizes the doping profile, nominal thicknesses, and layer sequence.

The device-fabrication sequence is summarized in Figure 2. The as-grown sample was first cleaned in acetone and isopropanol (5 min each), treated with oxygen plasma (3 min), and dipped in HCl:H_2_O (1:4) for 40 s to remove surface contamination and native oxide. The mesa and the exposed active region were then defined by UV photolithography with the positive resist S1818 (spin-coated at 4000 rpm with an acceleration of 2000 rpm/s for 30 s, soft-baked at 110 °C, exposed under UV light, and developed in AZ319), and the unmasked GaN layers were etched by inductively coupled plasma reactive-ion etching (ICP-RIE) with a Cl_2_/BCl_3_-based plasma chemistry to a target depth of 610 ± 50 nm. The electrode pattern was defined by a second photolithography step, and the Ti/Au (20/200 nm) contact stack was deposited by electron-beam evaporation followed by lift-off. Finally, Ti_3_C_2_T_x_ MXene was deposited in the grating slots by a further lithographic step, deposition of the MXene film, and lift-off, which completed the MXene-modified device. The process parameters were optimized to obtain well-defined mesas and a continuous grating pattern. As shown in Figure 1a, the dispersion was deposited by drop-casting onto the patterned device surface, and the MXene film was confined to the grating slots by the final lithographic lift-off step described above.

The colloidal Ti_3_C_2_T_x_ dispersion used in the deposition step described above was synthesized from commercial Ti_3_AlC_2_ MAX-phase powder (Foshan Xinxi Technology Co., Ltd., Foshan, China), used as received, as follows. LiF (3.2 g) and 40 mL of 9 M HCl were first reacted in a 100 mL polytetrafluoroethylene (PTFE) vessel at 40 °C for 15 min; Ti_3_AlC_2_ powder (2 g) was then added slowly under stirring at 500 rpm and the mixture was reacted at 40–45 °C for 48 h. The etched product was washed by repeated centrifugation (3500 rpm, 5 min, 7–8 cycles) with deionized water until the supernatant reached pH ≈ 6 and was then vacuum-filtered. Delamination was performed by redispersing the washed solid in deionized water (50 mL per tube) under an inert-gas atmosphere in an ice-bath sonicator for 1 h; after centrifugation at 3000 rpm for 30 min, the dark-green supernatant was collected as the few-layer Ti_3_C_2_T_x_ dispersion. The dispersion used for deposition had a concentration of 10 mg/mL in deionized water. The dispersion was drop-cast onto the patterned device area and dried on a hot plate at 60 °C for 10 min; the film was then confined to the grating slots by the final lift-off step described above. The SiO_2_ layer shown in Figure 2 was deposited by plasma-enhanced chemical vapor deposition (PECVD) to a thickness of 100 nm on the mesa-patterned wafer, before the Ti/Au metallization, and was patterned by anisotropic reactive-ion etching (RIE) in a CF4 plasma. It acts both as a passivation layer, protecting the etched mesa sidewalls and the field region, and as an insulation layer that separates the metallization from the surrounding field region. The RIE openings define the grating slots, in which the GaN surface remains exposed. The Ti/Au (20/200 nm) grating electrode was then formed by electron-beam evaporation and lift-off. Because the SiO_2_ layer is retained in the finished device, the grating slots remain open down to the GaN, and the Ti_3_C_2_T_x_ film deposited in the following step fills these slots and is in direct contact with the GaN surface rather than with the Ti/Au metallization.

An exposed, line-patterned central section connected to larger metal-contact portions is present in the finished structure. In this configuration, the metal pattern provides electrical access for current-voltage (I–V) measurements, while the exposed grating slots receive Ti_3_C_2_T_x_ MXene coverage; the MXene film is confined to these slots and does not extend over the metal contacts or the mesa. The patterned grating provides the optical access path, while the metal contacts provide the probe-contact and carrier-collection routes; the 254 nm beam can reach the active surface through the grating openings.

### 2.2. Morphological Characterization

The continuity and coverage of the deposited film were inspected by optical and scanning electron microscopy; complementary characterization of the MXene film by X-ray diffraction (XRD), X-ray photoelectron spectroscopy (XPS), energy-dispersive X-ray spectroscopy (EDS) mapping, Auger electron spectroscopy (AES), and atomic force microscopy (AFM) was performed to examine its structure, composition, surface chemistry, and topography, and the film thickness was measured with a step profiler; the results are presented in Section 3.1.

The finished device, including the contact architecture, grating continuity, and the exposed central region, was examined using optical microscopy and field-emission scanning electron microscopy (FESEM, ZEISS SUPRA-55, Carl Zeiss Microscopy GmbH, Jena, Germany), which confirmed the integrity of the grating pattern and the continuity of the metal contacts. The as-deposited Ti_3_C_2_T_x_ MXene film was inspected with the same tools to verify its coverage on the grating region.

### 2.3. Electrical Characterization

Static current-voltage (I–V) characteristics recorded in the dark and under 254 nm illumination from a UV LED were used for the electrical characterization. The measurements were performed in a two-terminal configuration using a Keithley 4200-SCS semiconductor parameter analyzer, with the device biased from −2 to +5 V in steps of 0.1 V (71 points per sweep). The dark measurements were carried out in a shielded enclosure to minimize ambient light and electrical interference, and the illuminated measurements were performed with the 254 nm LED beam incident on the exposed grating region. Illumination was provided by a 254 nm UV LED (3535 package with a quartz window). The emission of the LED was characterized with a spectroradiometer: at a drive current of 150 mA (forward voltage 6.162 V, electrical power 0.924 W), the peak wavelength was 255.5 nm with a FWHM of 10.2 nm, and the total radiant flux was 9.3253 mW, of which 9.1668 mW (98.3%) was in the UVC band. The source is referred to as the 254 nm LED throughout this work. The LED was placed approximately 1 cm above the device so that the approximately circular illuminated spot (diameter 1.155 cm, area ≈ 1.047 cm^2^) covered the exposed grating region. Because the angular emission profile of the packaged LED was not mapped and no calibrated detector was placed at the sample plane, the irradiance actually reaching the device was not determined; the flux, the spot size and the source–device distance given above define the illumination geometry reproducibly, but their combination is not an absolute in situ power density. The LED drive was held at a fixed setting during each measurement; because no independent detector monitored the sample plane during the sweep, short-term optical-power stability was not quantified directly. Each sweep was recorded between −2 and +5 V in 0.1 V steps (71 points per sweep). Forward sweeps were recorded for all device states; double sweeps were additionally recorded for selected device states. The dark state was measured first, and the illuminated sweep was recorded after a stabilization interval of 10 min, which was used to reduce the influence of LED warm-up. The current compliance was set to 10 mA. The sweeps were performed in DC mode (pulse mode disabled) at normal sweep speed, with no inter-point delay and a 1 s hold before each sweep, which corresponds to an acquisition time of approximately 0.1 s per point. Current and voltage were measured at every point, with the current range set to automatic and the voltage range set to the best fixed range. The cathode was connected to the common ground unit and the anode was biased by the source-measure unit; the measurements were controlled with KTEI V9.0 software.

Paired measurements made on the identical grating-electrode device before and after Ti_3_C_2_T_x_ MXene coverage make up the electrical comparison. The same device before MXene coverage is denoted as pristine, whereas its state after coverage is denoted as MXene-modified.

I_ph_ = I_light_ − I_dark_, (1)

Equation (1) was used to determine the net photocurrent, and the ratio I_light_/I_dark_ was used to define the light-to-dark (L/D) ratio. These two quantities are the response metrics used throughout this work. The quantitative comparison is limited to the +1 to +5 V range used in the response-metric figure; within this range, the +1 V after-to-before photocurrent quotient is reported for completeness only, because the pristine net photocurrent at +1 V is very small.

In addition to the paired dark/illuminated sweeps of the original device, further dark I–V measurements were performed to establish the device-to-device reproducibility of the transport modification and to provide control structures. The dark I–V characteristics were recorded for the original grating-electrode device (device 1) and for two additional grating-electrode devices from the same batch (devices 2 and 3), each measured both before and after Ti_3_C_2_T_x_ MXene coverage, and for a control structure processed without the grating electrode. Every device state was recorded as a single forward sweep (−2 → +5 V, 71 points) and, for most states, additionally as a double sweep (−2 → +5 → −2 V, 142 points) under the same DC conditions, current compliance, hold time, and 0.1 V step size as described above; all of these additional I–V measurements were performed in the dark. The time-resolved photoresponse was measured on the original MXene-covered device at a fixed bias of +1 V while the 254 nm LED was switched on and off with on/off intervals of 10 s and 20 s, the current being sampled every 0.1 s for about 320 s and 380 s, respectively, with the same LED drive setting and geometry as used for the I–V sweeps.

## 3. Results and Discussion

### 3.1. Structural and Chemical Characterization of the MXene Film

Figure 3 summarizes the structural, morphological, and compositional characterization of the deposited Ti_3_C_2_T_x_ film. The structural evolution from the Ti_3_AlC_2_ precursor to the MXene was examined by X-ray diffraction (XRD). As shown in Figure 3a, the (002) reflection of the Ti_3_AlC_2_ precursor is located at 2θ = 9.50°, whereas after HCl/LiF etching the corresponding reflection shifts to 2θ = 5.72°; according to Bragg’s law with Cu Kα radiation (λ = 0.15406 nm), these angles correspond to interlayer spacings of 0.930 nm and 1.544 nm, respectively. The shift towards lower angle and the increase in interlayer spacing indicate expansion of the layered structure after removal of the Al atomic layers, which is consistent with the formation of Ti_3_C_2_T_x_. The surface morphology of the film was characterized by scanning electron microscopy (SEM). Figure 3b,c show images of the MXene-covered device region at different magnifications: the deposited film consists of an interconnected network of overlapping flakes, and the higher-magnification image reveals the characteristic lamellar stacking of two-dimensional sheets. The elemental composition of the same region was examined by energy-dispersive X-ray spectroscopy (EDS) and Auger electron spectroscopy (AES). The EDS maps in Figure 3d show the spatial distribution of Ti, O, Ga, and N with atomic percentages of 3%, 3%, 82%, and 12%, respectively; the four distributions are displayed in a single multi-element panel so that they can be compared directly within the same field of view. The Ti- and O-related signals are associated with the deposited MXene, whereas the dominant Ga and N signals originate from the underlying nitride layers that are also sampled by the micrometer-scale EDS probe. The AES spectrum in Figure 3e provides complementary near-surface elemental information from the device region. Atomic force microscopy (AFM) was used to evaluate the surface topography (Figure 3f); the measured average roughness is Ra = 3.340 nm and the root-mean-square roughness is Rq = 5.136 nm, and the thickness of the deposited film was measured to be 29.8 nm (0.0298 μm) using a step profiler.

The surface chemical states of the deposited film were analyzed by X-ray photoelectron spectroscopy (XPS). The survey spectrum in Figure 4a shows Ti, C, O, F, and Cl, with atomic percentages of 27.52%, 36.41%, 18.39%, 13.03%, and 4.64%, respectively. No detectable Al signal was observed in the XPS survey spectrum, suggesting that the residual Al content in the analyzed near-surface region was below the detection limit under the present measurement conditions. The survey composition includes a substantial adventitious-carbon contribution, so these atomic percentages describe the surface composition of the deposited film rather than the stoichiometry of the MXene itself. High-resolution spectra of Ti 2p, C 1s, O 1s, F 1s, and Cl 2p were recorded and fitted (Figure 4b–f); the corresponding binding energies, FWHM values, relative peak-area fractions, and assignments are summarized in Table 1. The Ti 2p spectrum is dominated by Ti–C and Ti–C–F/X components with smaller Ti–C–O and Ti–O contributions; the C 1s spectrum comprises Ti–C–X, C–C, and C–O/OH–X components; the O 1s spectrum comprises Ti–O, Ti–C–O, Ti–C–OH, C–O/OH–X, and O–X components; the F 1s spectrum comprises Ti–C–F, F–X, and F–O–X components; and the Cl 2p spectrum comprises a single Ti–C–Cl doublet, tentatively assigned to a chlorine-containing surface chemical environment of the MXene [19].

Taken together, the high-resolution spectra indicate that the etched product has mixed oxygen-, fluorine-, and chlorine-containing surface chemical environments rather than a single termination type. These assignments identify element-containing chemical environments but do not, by themselves, establish specific covalent termination structures. A high-resolution Al 2p spectrum was not obtained: in the fabricated device the AlGaN layer is buried beneath the Ti_3_C_2_T_x_ film and the GaN-related layers, and the corresponding photoelectron signal is strongly attenuated under the present measurement conditions. The O 1s component at ca. 533.1 eV is attributed to C–O/OH species, including adventitious carbon and adsorbed water; this assignment is consistent with the C 1s component at ca. 286.4 eV.

### 3.2. Device Structure and Morphology

The finished grating-electrode device is depicted in Figure 5. The dense parallel line pattern and the large contact area are visible in the optical image. The central grating zone is formed over a wide area and remains connected to the surrounding contact pattern, as confirmed by scanning electron microscopy.

### 3.3. Static Dark and 254 nm Current-Voltage Characteristics

The device’s dark and 254 nm I–V characteristics before and after Ti_3_C_2_T_x_ MXene coverage are contrasted in Figure 6. A microampere scale is used to display the before-coverage state, whereas a milliampere scale is used to display the after-coverage condition. At positive applied voltages, both states show nonlinear, asymmetric transport and a discernible separation between the illuminated and dark curves. As a result, the light–dark separation, which indicates a light-induced current change under the tested conditions, is analyzed independently for each state and separately from the change in the absolute current level.

The nonlinear and polarity-dependent I–V characteristics are consistent with non-ideal carrier injection through an asymmetric surface-contact network. In a two-terminal grating device, unequal effective barriers, contact areas, or local series resistances can produce a back-to-back contact response in which the reverse-biased junction limits the current [21]. The present room-temperature curves alone cannot identify the dominant transport mechanism, which would require temperature-dependent I–V and independent barrier-height measurements. The curves in Figure 6 and Figure 7 connect the measured data points as guides to the eye; no functional fit was used to extract device parameters in the present static analysis.

The dark and illuminated currents at +3 V prior to MXene coverage were 1.98 and 3.88 μA, respectively, resulting in a net photocurrent of 1.90 μA. The dark and illuminated currents were 0.837 and 1.672 mA under the same bias following MXene coverage, while the net photocurrent was 0.835 mA. Therefore, both before and after Ti_3_C_2_T_x_ MXene coverage, the device offers an electrical criterion for differentiating between the illuminated and dark states at 254 nm.

### 3.4. Configuration-Dependent Transport

The identical paired measurements are arranged by operating condition in Figure 7. Figure 7a’s dark-current comparison reveals a greater current following Ti_3_C_2_T_x_ MXene coverage compared to prior coverage. The same current-scale ordering under illumination is seen in the comparison of Figure 7b at 254 nm. Taken together, the two panels demonstrate that Ti_3_C_2_T_x_ MXene coverage raises the device’s total transport level in both electrical states; the insets (yellow arrows) magnify the pristine-device curves, whose magnitude is too small to be resolved on the common scale.

At +3 V, MXene coverage increases both the dark current and the current under 254 nm illumination by broadly comparable orders of magnitude; for the representative pair shown, the light-to-dark ratio changes only modestly, from 1.96 to 2.00. Because Section 3.7 shows that the after-to-before quotient of the dark current ranges from about 4 × 10^2^ to 1.3 × 10^3^ depending on the device and the sweep type, and from about 500 to about 1250 for device 1 at +3 V depending on the sweep used, the two quotients obtained from this single measurement pair are not constrained well enough to support a quantitative comparison. The more robust observation is therefore that both electrical states are enhanced by a similarly large factor. This overall scaling is more consistent with a large increase in the effective conductance of the measured path than with a selective increase in photocarrier generation or separation. Possible contributions include a parallel MXene conduction path, reduced effective contact resistance, barrier lowering, and increased surface carrier density. These possibilities cannot be separated using the present static I–V data. The MXene film is confined to the grating slots, where it is in direct contact with the exposed GaN, and it does not extend over the metal contacts or the mesa (Section 2.1); a direct MXene bridge over the metal-contact-to-mesa region is not expected from the designed geometry. Lateral conduction within the MXene-covered grating region cannot be separated from a reduction in the effective contact resistance or from barrier modulation, and the present static data cannot exclude any of these contributions.

### 3.5. Net Photocurrent and Light-to-Dark Ratio from +1 to +5 V

The two derived response metrics, which were computed from the device’s light and dark I–V curves before and after Ti_3_C_2_T_x_ MXene coverage, are shown in Figure 8. Plotting the net photocurrent on a logarithmic scale, Figure 8a demonstrates that I_ph_ rises with applied voltage in both states and remains higher after MXene coverage. Table 2 summarizes the representative +1 to +5 V data points used for the quantitative comparison. The net photocurrent and the light-to-dark ratio are the relative response metrics adopted here, following the common practice for photodetector characterization [21]; the absolute figures of merit are discussed below.

It should be noted that absolute figures of merit for photodetectors are not reported in the present study because the radiant flux of the 254 nm source was characterized separately (Section 2.3) whereas the power actually incident on the device was not measured with a calibrated power meter at the sample plane [21]. The relative metrics Iph and the L/D ratio are therefore used to compare the pristine and MXene-modified states of the same device. In addition, the net photocurrent is obtained as the difference between two currents that differ by only a factor of about two, so any responsivity derived from these data would carry a large relative uncertainty and would not be a reliable absolute figure of merit. A direct quantitative comparison with previously reported MXene/GaN detectors [9,10,11,12] is likewise limited because those studies typically report calibrated responsivity and detectivity values obtained under different illumination and bias conditions. Calibrated power-dependent measurements and noise characterization would be required to extract absolute figures of merit; quantifying absolute detector performance is beyond the scope of the present static transport study.

I_ph_ is equal to I_light_ minus I_dark_. The illuminated-to-dark current ratio is represented by L/D. M/P is not a detector-performance improvement factor; rather, it is the absolute after-to-before photocurrent quotient for the pristine and MXene-modified states of the same device. Since the net photocurrent prior to MXene coverage is nearly nil, the +1 V quotient is reported for completeness but is not utilized as a typical value. Given the device-to-device and sweep-to-sweep variability reported in Section 3.7, these M/P values should not be read as a precise enhancement factor.

### 3.6. Working Mechanism and Static Light–Dark Current Separation

With an energy of about 4.88 eV, a 254 nm photon exceeds the GaN bandgap and can therefore generate photocarriers not only in the quantum wells but also in the n^+^-GaN contact layers and potentially in the AlGaN barriers. Before being collected by the Ti/Au electrodes, these carriers travel through the epitaxial stack, the n^+^-GaN contact layer, and the patterned surface-contact area under applied bias. The carrier-collection geometry and the exposed optical region are defined by the grating electrode; because the generation volume is not spatially localized, the static two-terminal I–V measurements cannot by themselves attribute the photoresponse to the coupled quantum wells or establish a polarization-field-enhanced carrier separation.

Through interfacial charge transfer, work-function-dependent barrier modulation, carrier-density changes, or contact-resistance changes, Ti_3_C_2_T_x_ MXene can affect the surface transport pathway [9,10,11,12]. A cautious interpretation that Ti_3_C_2_T_x_ MXene primarily modifies the overall transport level is supported by the paired data’s simultaneous rise in dark current and net photocurrent following MXene coverage. The observed separation between the dark and 254 nm-illuminated currents under an applied voltage demonstrates that the two electrical states can be distinguished, independent of the transport-level change; this corresponds to a static light–dark current separation rather than a quantified detection figure of merit.

A qualitative interface picture is therefore proposed in Figure 9 rather than a fitted band diagram. When Ti_3_C_2_T_x_ contacts the exposed n^+^-GaN surface, equilibration of the two Fermi levels can redistribute interfacial charge and modify local band bending. Mixed surface terminations, adsorbates, oxidation, and GaN surface states may shift the effective work function and barrier height [17,18]; these interface effects would require contact-resistance analysis (for example, a transfer-length method) and temperature-dependent I–V measurements to identify the dominant mechanism. Under illumination, photocarriers generated in the GaN/AlGaN structure are collected through this modified contact network.

Polarization discontinuities at the wurtzite GaN/Al_0.25_Ga_0.75_N interfaces can also contribute to the internal band profile of the asymmetric coupled quantum wells [5,6]. Their quantitative contribution in the present device depends on the actual layer composition, strain, doping, and electrostatic screening and should therefore be obtained from a self-consistent Poisson–Schrodinger calculation rather than from a bulk spontaneous-polarization value alone. The work-function values shown schematically in Figure 1 are not treated as direct measurements of the present MXene/n^+^-GaN interface. Because no direct work-function or barrier-height measurement was performed in this study, the effective barrier height and the direction and magnitude of band bending remain undetermined.

### 3.7. Device-to-Device Reproducibility and Control Structures

To establish whether the transport modification produced by Ti_3_C_2_T_x_ MXene coverage is reproducible, the dark I–V characteristics of the original grating-electrode device (device 1) and of two further grating-electrode devices from the same batch (devices 2 and 3) were measured before and after MXene coverage under identical conditions, together with a control structure processed without the grating electrode (Figure 10). In the pristine state, the three grating devices are closely grouped: at +5 V the single-sweep dark currents of devices 1–3 are 6.17, 6.29, and 6.72 μA, that is 6.39 μA on average with a device-to-device spread of ±4.5%, and the forward branches of the double sweeps of the same states give 6.60, 6.25, and 6.24 μA, i.e., within 8% of the single sweeps. All curves retain the same nonlinear, polarity-dependent shape. After MXene coverage the same three devices carry 5.09, 6.63, and 6.20 mA at +5 V in the single sweeps, which corresponds to an increase by factors of 826, 1055, and 923 relative to the pristine value of the same specimen. The forward branches of the double sweeps give appreciably lower post-coverage currents (2.76 and 4.91 mA for devices 1 and 2), so that the corresponding quotients are 418 and 785; for the MXene-covered state the current measured in a single forward sweep is thus 1.3–1.9 times higher than that measured in the forward branch of a double sweep recorded a few minutes earlier. The after-to-before quotient of the dark current therefore lies between about 4 × 10^2^ and 1.3 × 10^3^ depending on the device and the sweep type, which is somewhat larger than, but of the same order as, the value of about 423 obtained from the paired light/dark session of the original device (Section 3.4); the two sessions agree to within a factor of about three. Accordingly, the reproducibility of the MXene-covered state is poorer than that of the pristine state, for which the single and double sweeps agree to within 8%. Because the additional lithography, solvent exposure, lift-off, drying, and renewed probing were not applied to a matched no-MXene control, contributions from the processing history cannot be fully excluded.

The control structure without the grating electrode (six sweeps) shows a dark current of 8.34 ± 1.63 μA at +5 V, that is of the same order as, and about 1.3 times higher than, the 6.38 ± 0.22 μA obtained from all six sweeps of the pristine grating devices; the grating pattern alone therefore does not produce the milliampere-level currents of the MXene-covered devices. Taken together, these measurements show that the increase in the dark current by two to three orders of magnitude caused by MXene coverage is reproduced on all three devices, so that it is a device-level effect rather than a property of a single specimen. They also define the limits of the present statistics: the illuminated I–V characteristics of devices 2 and 3 were not recorded, so the photoresponse data of Section 3.5 and Section 3.8 remain those of the original device, and the run-to-run variation in the MXene-covered state is comparable to its device-to-device variation, so that no uncertainty better than a factor of about two can be assigned to the post-coverage current level.

Because most device states were also recorded as a double sweep, the two sweep directions can be compared directly (Figure 10d). The two branches converge at +5 V, where the reverse branch differs from the forward branch by only 0.1–1.1% on the pristine and grating-free devices and by 2–6% on the MXene-covered devices. Away from +5 V the reverse branch is systematically lower in magnitude than the forward branch in all eight double sweeps. For the pristine grating-electrode devices the reverse current falls to 10–62% of the forward current between +0.5 and +4 V, the ratio decreasing monotonically towards low bias (about a factor of five at +0.5 V and a factor of two to three at +2 to +3 V); the grating-free devices show a milder asymmetry (40–97%), whereas the MXene-covered devices show a nearly uniform 9–17% reduction over the same range. In the reverse-bias region, the reverse branch lies 0.6–55% closer to zero on the pristine and grating-free devices and 6–28% closer on the MXene-covered devices. Sweep-direction hysteresis is therefore not negligible in the present device: it is systematic and reproducible, it is largest where the forward current is still small, and it also affects the after-to-before quotient, which for device 1 at +3 V varies from about 500 to about 1250 depending on the sweep used. For this reason, the single-sweep values and the forward branches of the double sweeps are quoted separately throughout this work rather than being averaged, and a current quoted at a given bias should be qualified by the sweep direction.

### 3.8. Time-Resolved Light-On/Off Response

Figure 11 shows representative segments of the current recorded from the original MXene-covered device at a fixed bias of +1 V while the 254 nm LED was switched on and off with intervals of 10 s and 20 s. The summary values below were calculated from the full 316 s and 376 s records. The current alternates between two well-defined levels and follows the switching events in the displayed segments without an evident delayed transition at the available sampling interval. For the 10 s chopping, the dark level is 0.245 mA and the illuminated level 0.527 mA, giving a net photocurrent of 0.282 mA and a light-to-dark ratio of 2.15 over 16 on/off cycles (316 s); for the 20 s chopping the corresponding values are 0.229 mA, 0.480 mA, 0.251 mA, and 2.10 over nine complete cycles (376 s). Both ratios agree with the value of 2.16 obtained at the same bias from the paired I–V sweeps of the same device state (Table 2), so the static light–dark separation is reproduced in a time-resolved measurement. The observed current transitions were not resolved at the approximately 0.1 s sampling interval. Consequently, the intrinsic rise and decay times could not be accurately determined under the present measurement conditions. The plateau levels are not perfectly constant: the illuminated level decays by 12.5% (10 s chopping) and 6.5% (20 s chopping) between the first and the last cycle, and the dark level by 8.6% and 4.1%, respectively. This slow monotonic drift of a few percent per minute does not, however, change the light-to-dark contrast, which remains within 2.06–2.29 in every cycle of both records. Because the ratio is stable over a record lasting more than five minutes, the drift observed over the 10 min stabilization interval is expected to be on the order of several percent; its exact effect on the reported light-to-dark ratio is not rigorously bounded by the present record. The dark plateaus are quiet (standard deviation 0.17–0.35 μA, below 0.2% of the dark current), whereas the illuminated plateaus are noisier (1.7–2.5 μA), so the measured contrast is limited by drift rather than by noise. These measurements supply the repeated light-on/off cycles, an upper bound on the switching times, and a short-term stability record that static I–V data cannot provide; they do not constitute a calibrated responsivity measurement, because the optical power incident on the device was still not measured with a power meter.

Scope and limitations. This work should be regarded as a preliminary transport study centered on static I–V measurements, with limited time-resolved data from one device. The dark I–V characteristics were reproduced on three grating-electrode devices from the same batch and on a grating-free control structure, and the MXene-induced increase in the dark current was reproduced on all three devices, the after-to-before quotient lying between about 4 × 10^2^ and 1.3 × 10^3^ (Section 3.7); the illuminated I–V characteristics, the light-to-dark contrast, and the time-resolved response were, however, obtained from the original device only (n = 1), so the photoresponse statistics remain those of a single specimen. Static I–V data cannot distinguish a parallel MXene conduction path within the grating region, reduced effective contact resistance, or barrier modulation; because the MXene film is confined to the grating slots (Section 2.1), these contributions are restricted to the grating region and cannot be separated from one another. In addition, the lithography, solvent exposure, lift-off, drying, and renewed probing associated with MXene integration were not applied to a matched no-MXene control, so a contribution from the processing history cannot be excluded. Time-resolved photoresponse was recorded only over a few minutes at a single bias (Section 3.8), and spectral selectivity and long-term environmental stability were not measured, so the possible introduction of trap states, a change in the UV spectral selectivity, or degradation of the MXene by oxidation during the measurements cannot be assessed. Because the optical power incident on the device was not calibrated, absolute figures of merit are not reported, and a quantitative comparison with literature MXene/GaN photodetectors is not attempted. The conclusions are therefore limited to the demonstration of static light–dark current separation and transport modification in the same device under 254 nm illumination; calibrated power-dependent measurements, spectral response, temperature-dependent I–V, and device-to-device photoresponse statistics are required to quantify detector performance.

## 4. Conclusions

Ti/Au (20/200 nm) contacts were used to create a grating-electrode GaN/AlGaN asymmetric coupled-quantum-well device with a Ti_3_C_2_T_x_ MXene-modified surface region. Microscopy images verify the patterned contact and grating structure, and the fabrication sequence explains the integration procedure. Before and after Ti_3_C_2_T_x_ MXene coverage, paired static I–V measurements on the same device reveal nonlinear transport and discernible light-induced current changes under 254 nm illumination in both states. At +3 V, the light-to-dark ratios are 1.96 and 2.00, and the net photocurrents before and after coverage are 1.90 μA and 0.835 mA. The increase in the dark current was reproduced on three grating-electrode devices (approximately 400× to 1300× relative to their own pristine values, depending on the device and the sweep direction), whereas a control structure without the grating electrode carried a dark current nearly three orders of magnitude below that of the MXene-covered devices. Switching the 254 nm source at a fixed bias of +1 V produced reproducible switching between two current levels with a light-to-dark ratio of 2.10–2.15, transitions completed within the 0.1 s sampling interval, and a drift of a few percent per minute without loss of contrast. Ti_3_C_2_T_x_ MXene coverage mostly raises the overall transport level while maintaining a detectable relative light–dark contrast, as indicated by the similar order of magnitude of the dark-current and net-photocurrent increases. Within the scope of this study centered on a single-device paired static comparison, the paired light–dark electrical measurements demonstrate static separation between the dark and 254 nm-illuminated states and modification of the overall transport level by MXene coverage; an improvement in photodetector performance is not claimed, and absolute figures of merit are not reported because the optical power incident on the device was not calibrated.

The present evidence does not determine the microscopic origin of the conductance change. Future work should combine temperature-dependent transport with UPS/XPS or Kelvin-probe measurements, contact-resistance analysis (e.g., transfer-length method), and extended time-resolved photoresponse on multiple devices. These measurements would test whether the observed current increase originates mainly from work-function-dependent barrier modulation, a parallel MXene path, changes in GaN surface states, or a combination of these effects. In parallel, calibrated power-dependent measurements, noise characterization, and device-to-device statistics are required before any absolute detector-performance claims can be made. The elemental composition and coverage of the MXene film were examined by EDS mapping and XPS survey spectra, and its surface morphology by SEM and AFM (Section 3.1). Raman spectroscopy was not performed in the present study and is not used to support the structural assignment.

## Figures and Tables

**Figure 1:**
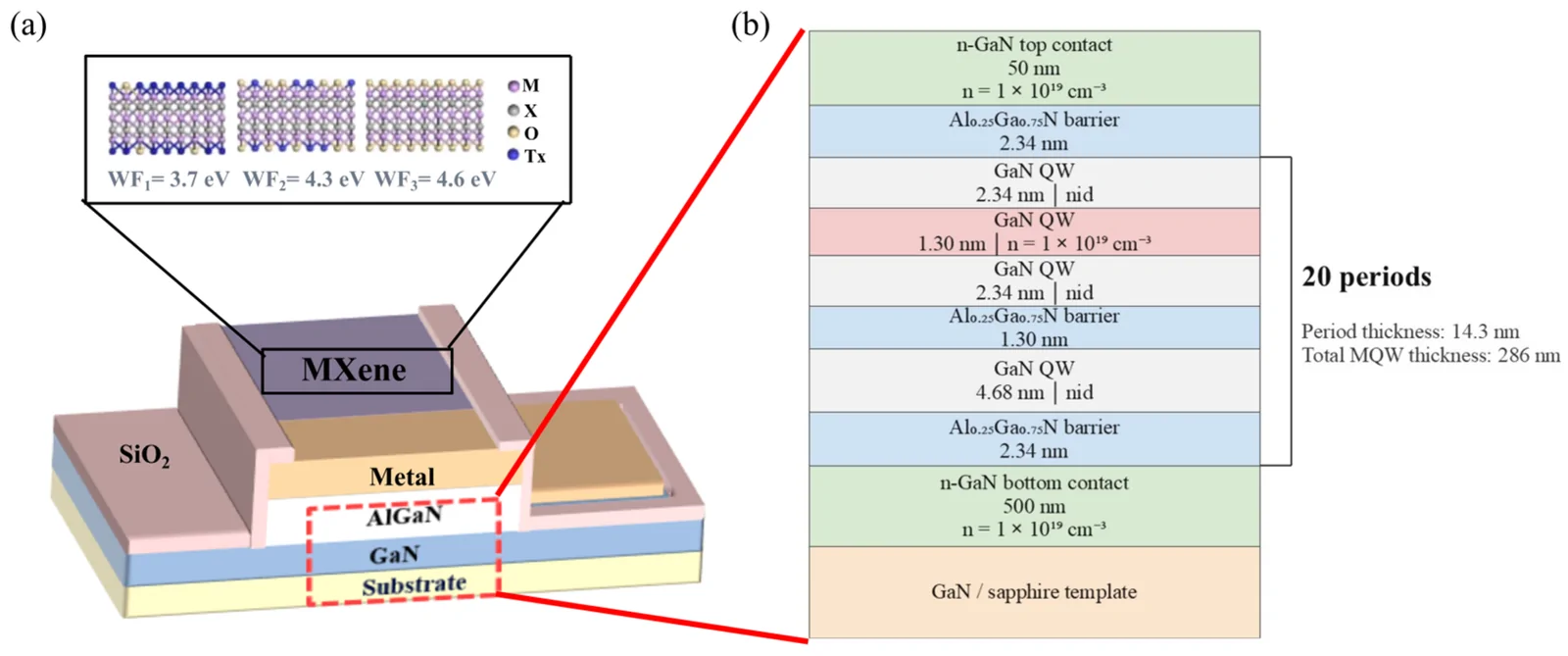
(**a**) Schematic diagram of the MXene-modified grating-electrode GaN/AlGaN device; the work-function values in the inset are schematic values and were not experimentally measured for the present interface. (**b**) Schematic epitaxial structure of the 20-period GaN/AlGaN asymmetric coupled-quantum-well device.

**Figure 2:**
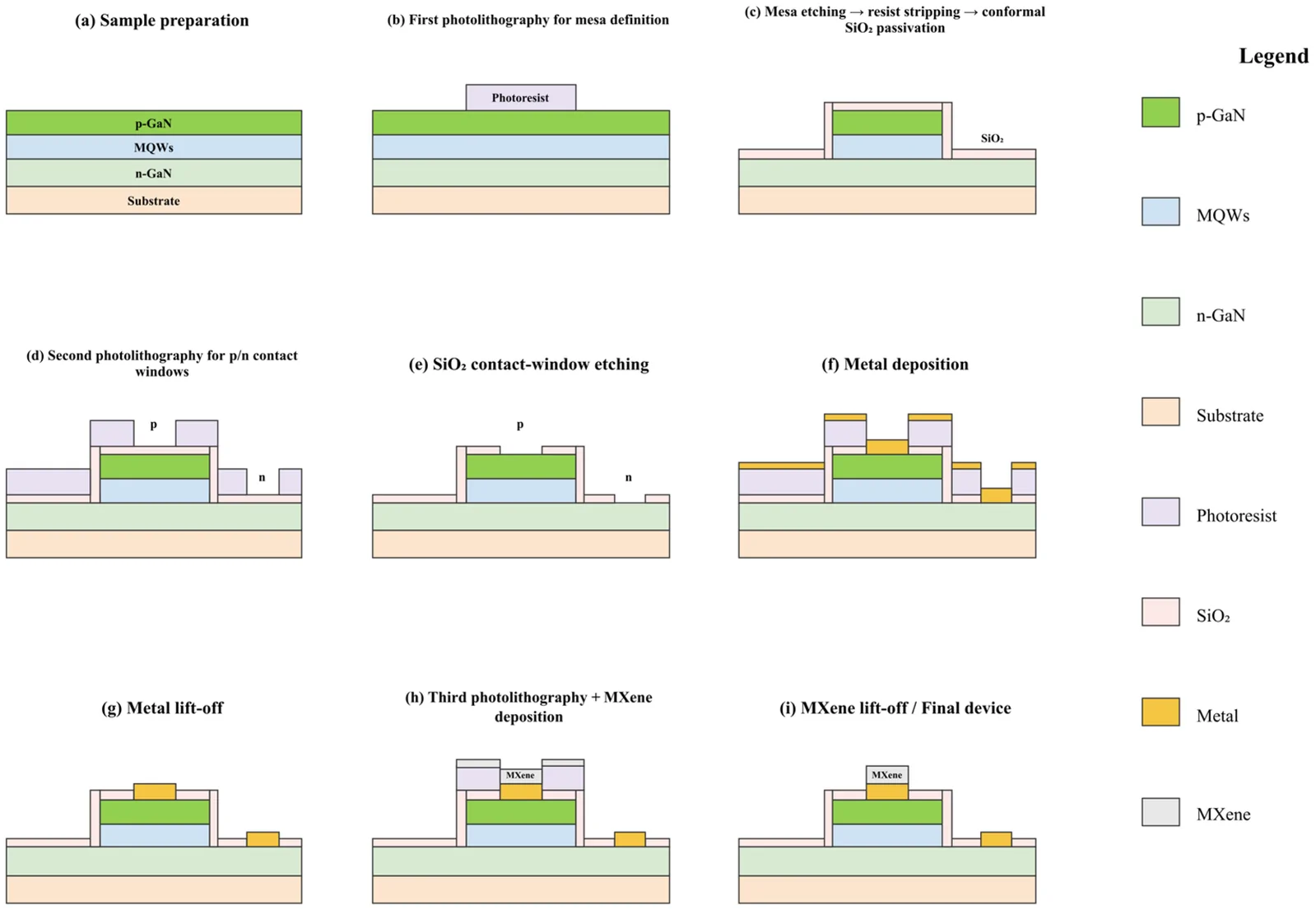
Schematic fabrication sequence of the Ti_3_C_2_T_x_ MXene-modified grating-electrode GaN/AlGaN quantum-well photodetector.

**Figure 3:**
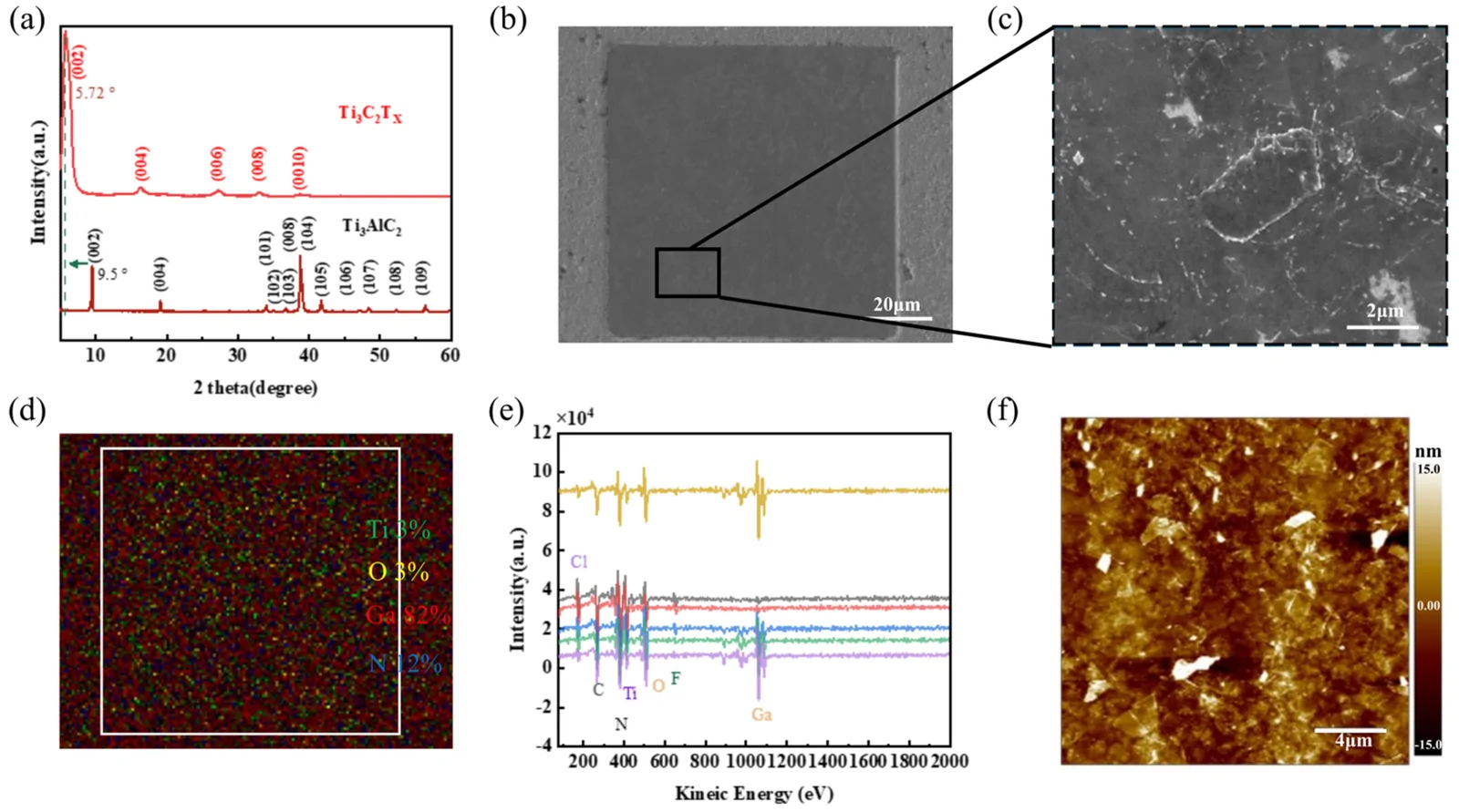
Structural, morphological, and compositional characterization of the Ti_3_C_2_T_x_ MXene film. (**a**) XRD patterns of the Ti_3_AlC_2_ precursor and of the Ti_3_C_2_T_x_ MXene. (**b**,**c**) SEM images of the MXene-covered device region at different magnifications. (**d**) EDS elemental maps of Ti, O, Ga, and N with the corresponding atomic percentages. (**e**) Auger electron spectroscopy (AES) spectrum of the selected device region. (**f**) AFM topographic image of the deposited film. Dashed outlines and connecting lines identify selected regions and their magnified views, and the colors in the EDS maps correspond to the elements labeled in panel (**d**).

**Figure 4:**
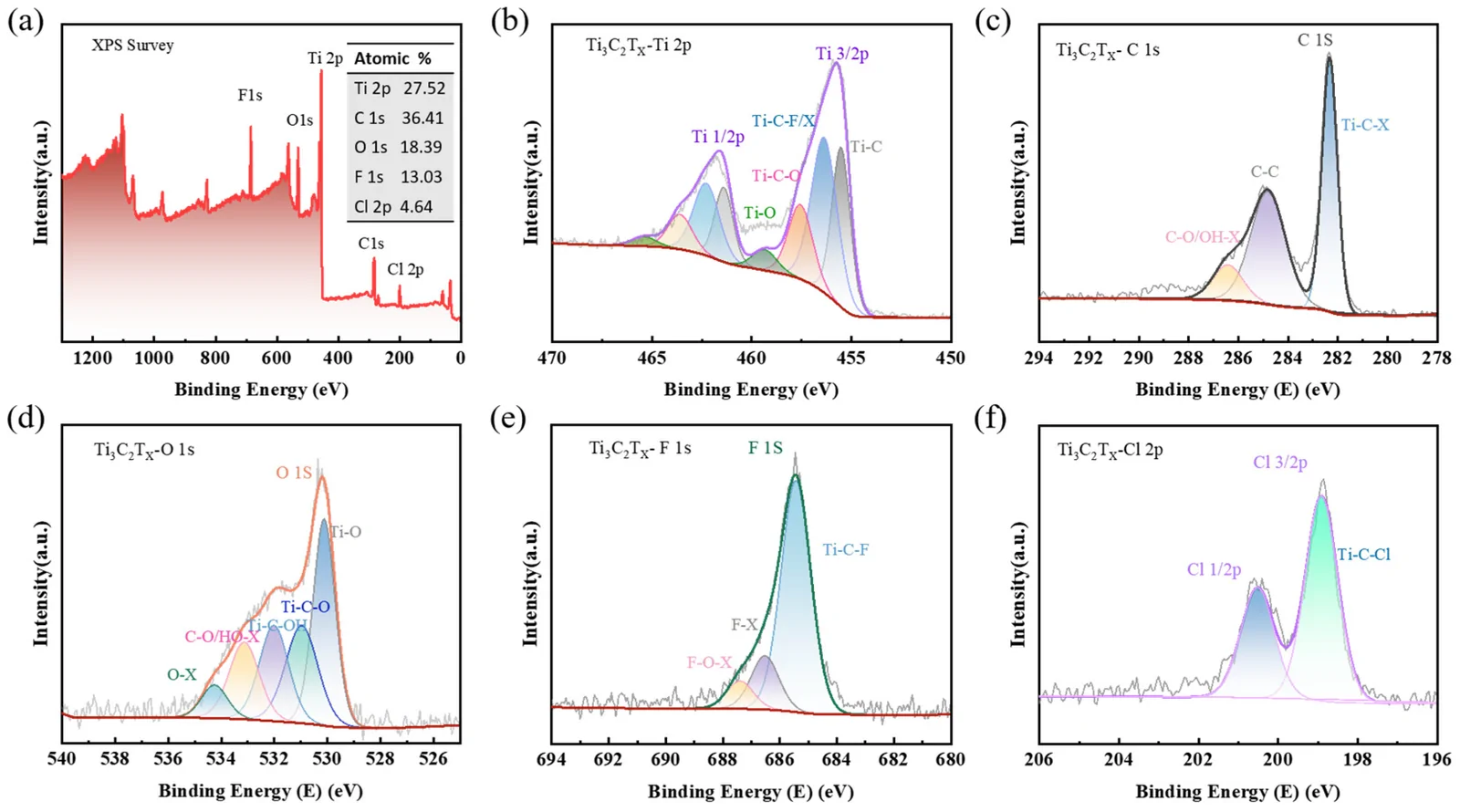
XPS characterization of the surface chemical states of the Ti_3_C_2_T_x_ MXene. (**a**) Survey spectrum with the corresponding atomic percentages. High-resolution spectra and the corresponding fits of (**b**) Ti 2p, (**c**) C 1s, (**d**) O 1s, (**e**) F 1s, and (**f**) Cl 2p. The fitting parameters are listed in Table 1.

**Figure 5:**
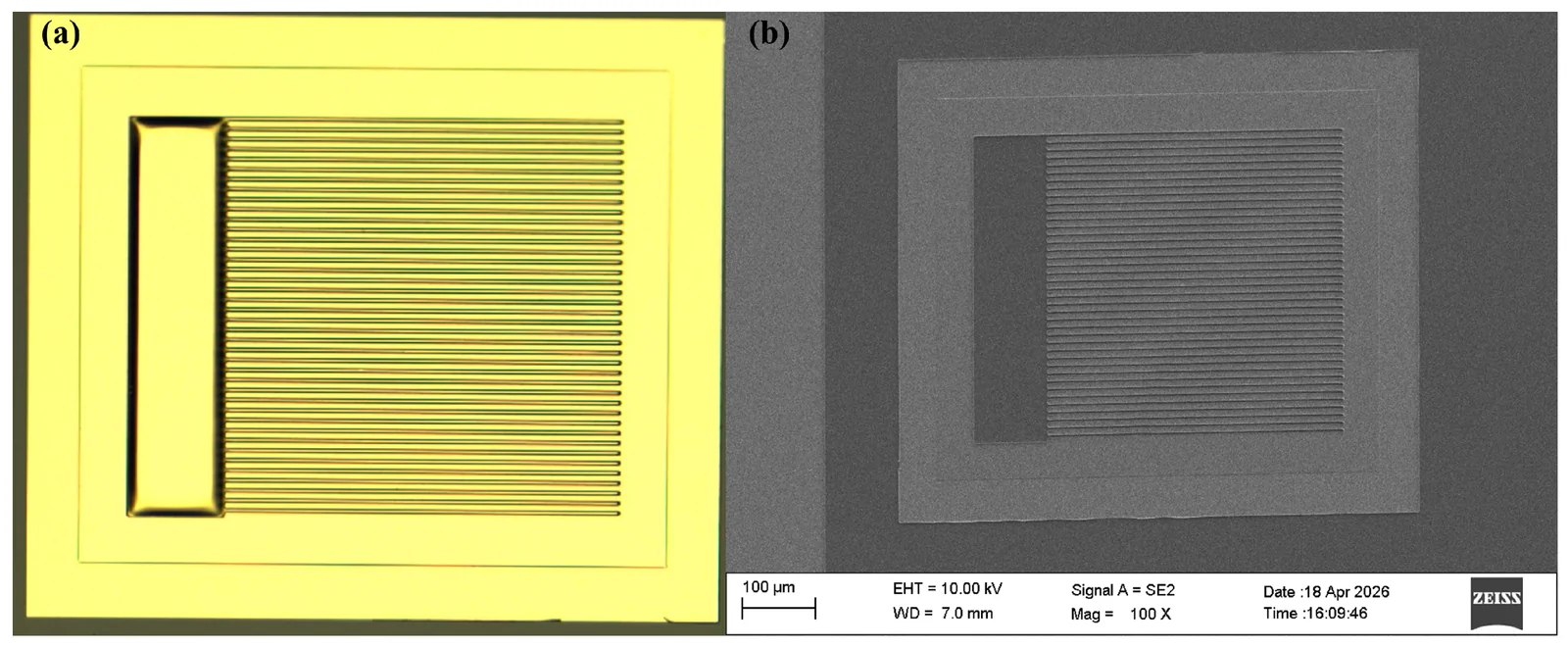
Completed grating-electrode device: (**a**) optical image; (**b**) scanning electron micrograph.

**Figure 6:**
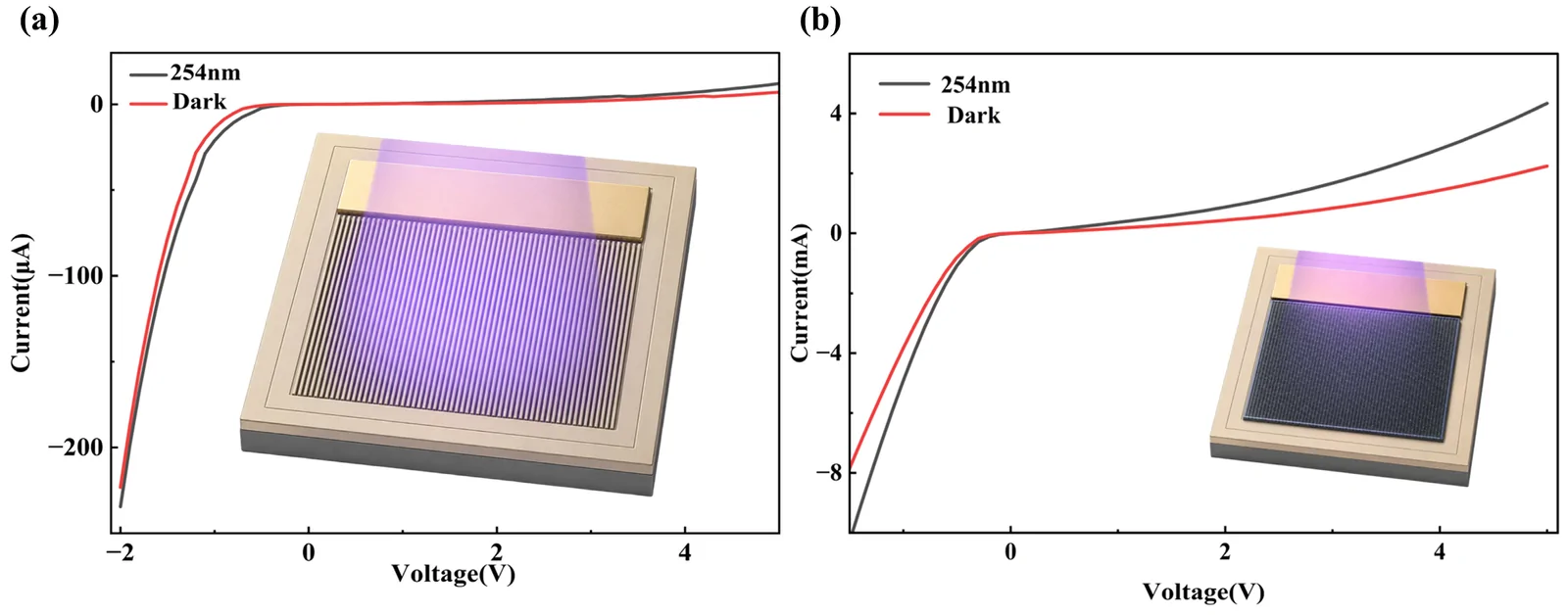
Dark and 254 nm I–V characteristics of the same grating-electrode device: (**a**) before Ti_3_C_2_T_x_ MXene coverage (pristine); (**b**) after Ti_3_C_2_T_x_ MXene coverage (MXene-modified). The current axes are in μA in (**a**) and mA in (**b**), and both panels share the same voltage axis. Separate scales are required because the current level increases by two to three orders of magnitude after MXene coverage; a common-scale comparison of the two states is given in Figure 7.

**Figure 7:**
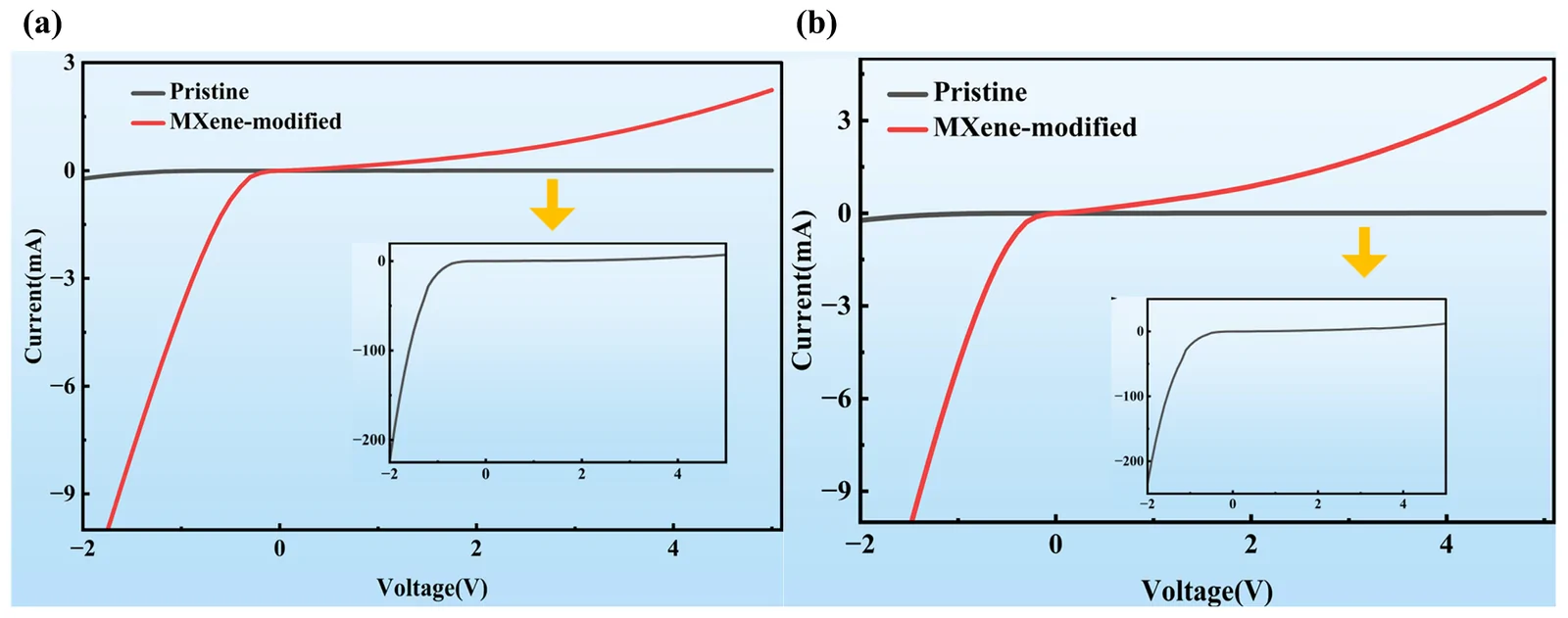
Paired comparison of the same grating-electrode device before and after Ti_3_C_2_T_x_ MXene coverage: (**a**) dark current; (**b**) current under 254 nm illumination. The main panels of both (**a**) and (**b**) plot the two states on a common current axis (in milliamperes, mA). The insets, identified by the yellow arrows, show magnified views of the pristine-device curves on an expanded scale (in microamperes, μA), because their current magnitude is much smaller than that of the MXene-modified curves.

**Figure 8:**
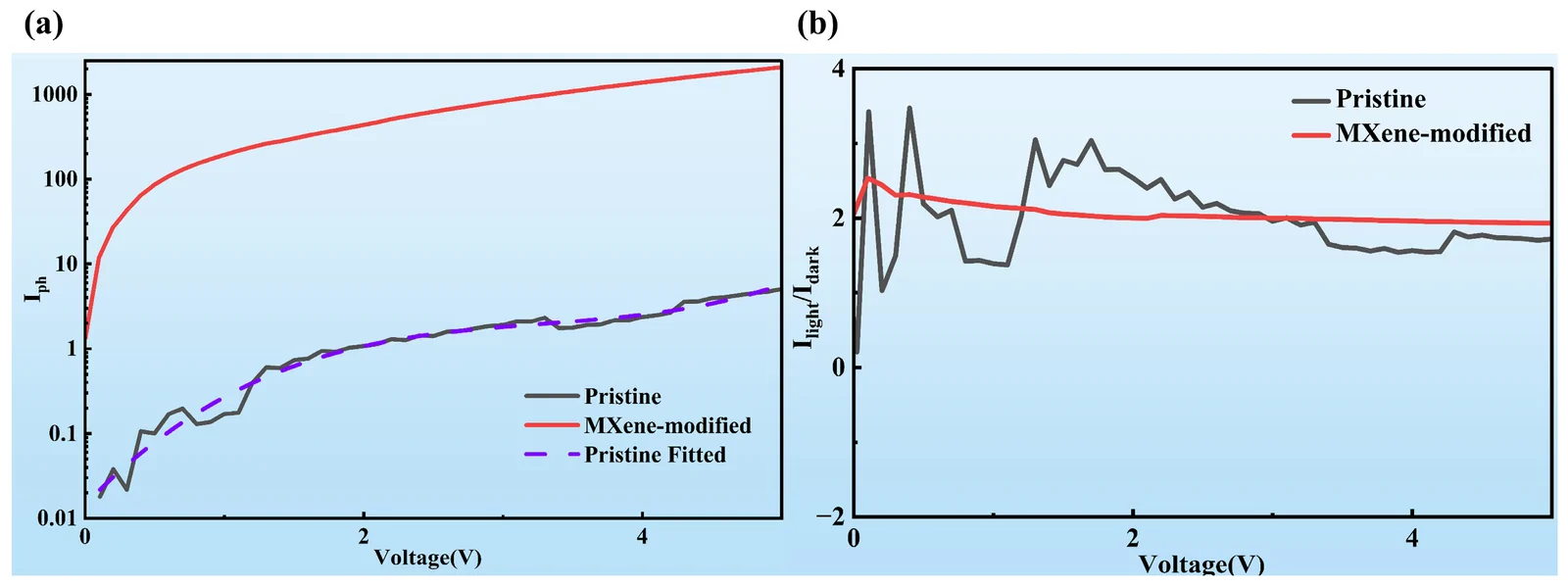
Derived response metrics of the same grating-electrode device before and after Ti_3_C_2_T_x_ MXene coverage: (**a**) net photocurrent as a function of applied voltage, plotted in microamperes on a logarithmic scale; (**b**) illuminated-to-dark current ratio as a function of applied voltage. Quantitative comparisons in the text and Table 2 use the +1 to +5 V interval.

**Figure 9:**
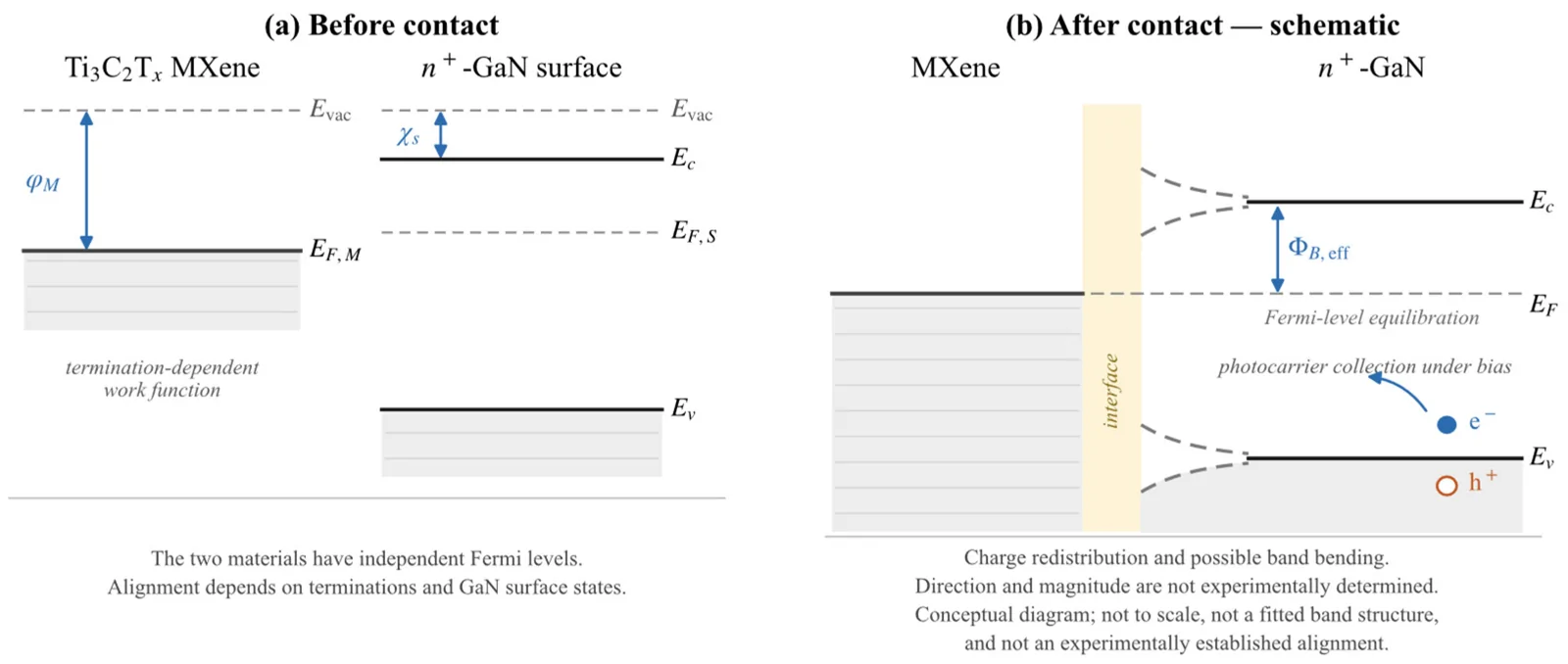
Qualitative schematic showing one possible scenario for the Ti_3_C_2_T_x_/n^+^-GaN contact in the MXene-modified grating device: (**a**) separated materials before contact; (**b**) after contact under bias, illustrating possible equilibrium band bending and an effective injection barrier, together with the qualitative carrier-collection process under applied bias. The direction and magnitude of the band bending are not experimentally determined because no direct work-function or barrier-height measurements were performed. This diagram is conceptual and not to scale; it represents only one possible qualitative scenario and does not imply an experimentally established band alignment, band offset, or barrier height.

**Figure 10:**
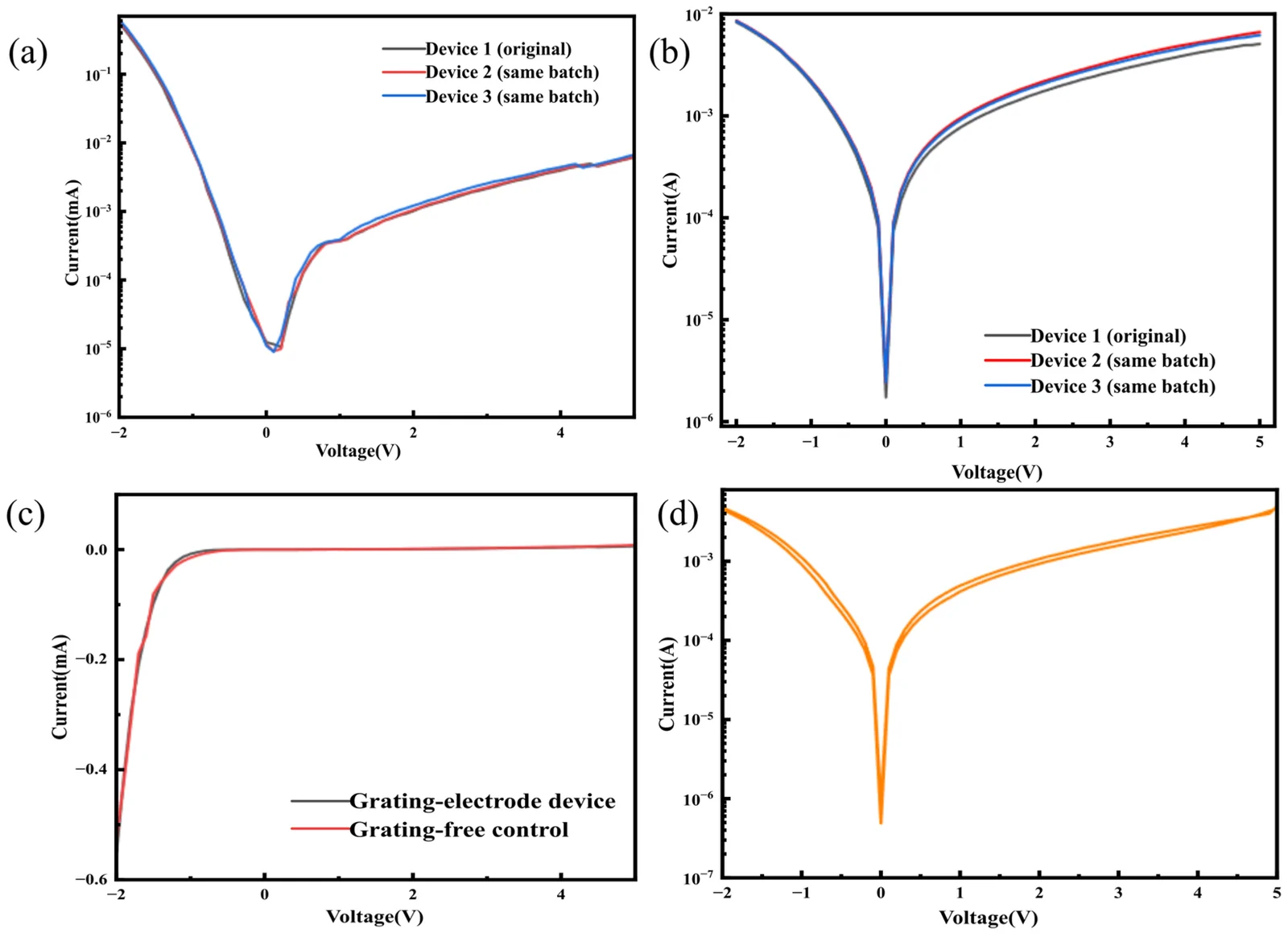
Device-to-device reproducibility of the MXene-induced transport modification and control structures. (**a**) Dark I–V characteristics of three grating-electrode devices (device 1, original; devices 2 and 3, from the same batch) before Ti_3_C_2_T_x_ MXene coverage (pristine), each recorded as a single forward sweep. (**b**) The same three devices after Ti_3_C_2_T_x_ MXene coverage (MXene-modified). The current magnitude |I| is plotted on a logarithmic scale in both (**a**,**b**), and the two panels share the same current-axis range so that the increase produced by MXene coverage can be compared directly between them; data points at which the current changes sign are not shown. (**c**) Comparison of the dark I–V characteristics of the pristine grating devices with those of the control structure without the grating electrode. (**d**) Comparison of the two sweep directions obtained from the double sweeps of the same device states, showing the systematic sweep-direction dependence discussed in the text. All measurements shown were performed in the dark.

**Figure 11:**
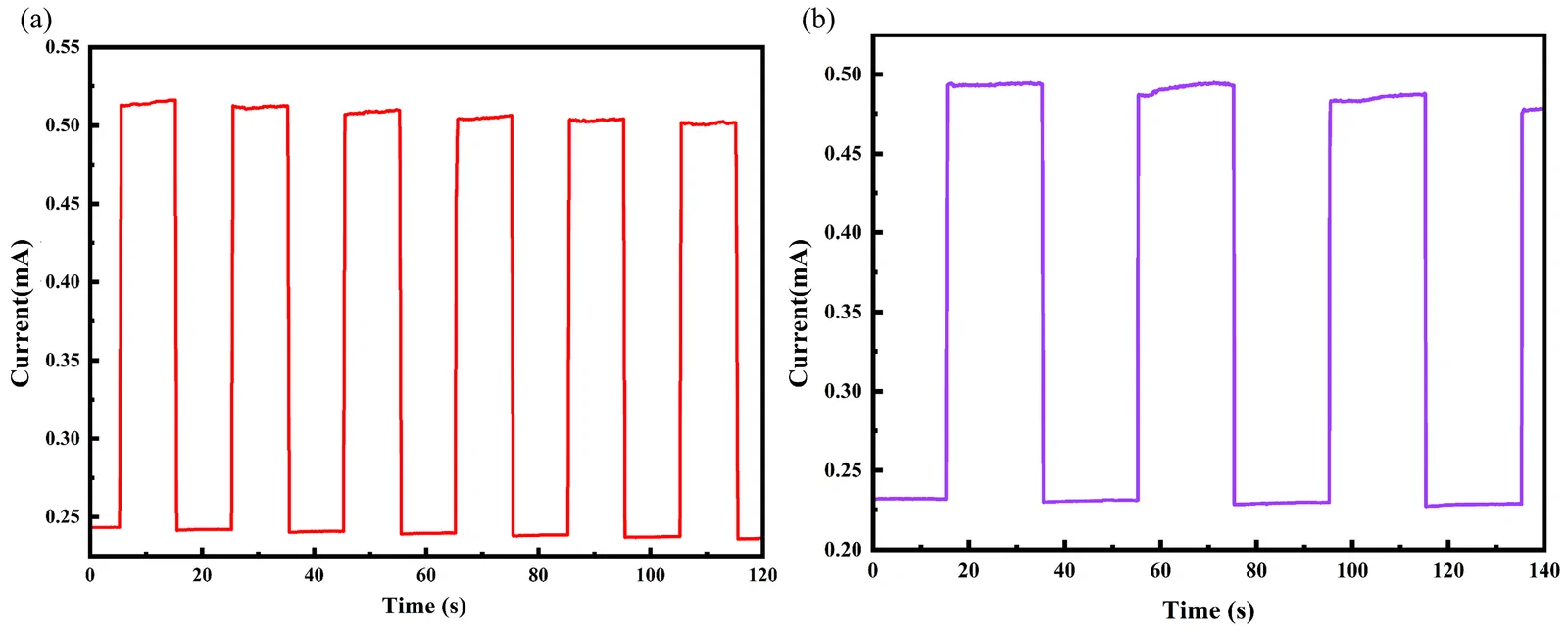
Representative segments of the time-resolved response of the original MXene-covered grating-electrode device at a fixed bias of +1 V, recorded while the 254 nm LED was switched on and off at intervals of (**a**) 10 s and (**b**) 20 s. Summary values in the text were calculated from the full 316 s and 376 s records. The insets emphasize the rise and decay edges, which are completed within the approximately 0.1 s sampling interval of the measurement.

**Table 1:** Fitting parameters of the high-resolution XPS spectra of the Ti_3_C_2_T_x_ MXene, including the binding energy (BE), the full width at half maximum (FWHM), the relative peak-area fraction, and the peak assignment.

Region	BE (eV)	FWHM (eV)	Fraction (%)	Assigned to
Ti 2p_3/2_ (2p_1/2_)	455.50 (461.39)	1.09	31.69	Ti–C
	456.35 (462.27)	1.53	43.34	Ti–C–F/X
	457.54 (463.59)	1.44	19.52	Ti–C–O
	459.34 (465.39)	1.44	5.46	Ti–O
C 1s	282.33	0.73	43.85	Ti–C–X
	284.80	1.65	43.97	C–C
	286.41	1.44	12.18	C–O/OH–X
O 1s	530.12	0.99	32.90	Ti–O
	530.96	1.44	22.67	Ti–C–O
	532.01	1.30	20.10	Ti–C–OH
	533.13	1.34	16.65	C–O/OH–X
	534.25	1.44	7.69	O–X
F 1s	685.43	1.23	75.38	Ti–C–F
	686.51	1.15	16.45	F–X
	687.39	1.11	8.17	F–O–X
Cl 2p_3/2_ (2p_1/2_)	198.86 (200.46)	1.02	100	Ti–C–Cl

The high-resolution spectra were fitted with Avantage 6.9.0 software. Binding energies were charge-corrected to the adventitious carbon C 1s peak at 284.8 eV. For the Ti 2p and Cl 2p spin–orbit doublets, the binding energies are given as 2p_3/2_ (2p_1/2_); X denotes an unresolved surface species. For the Cl 2p spin–orbit doublet, the spin–orbit separation was constrained to 1.60 eV and the component area ratio to 2:1 (Cl 2p3/2:Cl 2p1/2), with equal FWHM and equal Gaussian–Lorentzian character for both components; a Shirley background was applied. The main peak assignments are consistent with the NIST X-ray Photoelectron Spectroscopy Database [20].

**Table 2:** Paired comparison from +1 to +5 V of the same grating-electrode device before and after Ti_3_C_2_T_x_ MXene coverage under 254 nm illumination.

Bias (V)	Pristine I_ph_	MXene I_ph_	I_ph_ Quotient (M/P)	Pristine L/D	MXene L/D
+1	0.170 μA	193.859 μA	1140.10	1.39	2.16
+2	1.078 μA	437.184 μA	405.40	2.54	2.00
+3	1.901 μA	835.100 μA	439.31	1.96	2.00
+4	2.379 μA	1.380 mA	580.29	1.57	1.96
+5	5.067 μA	2.096 mA	413.79	1.72	1.93

## Data Availability

The data supporting the findings of this study are available from the corresponding author upon reasonable request.
